# Household Transmission of Mpox to Children and Adolescents, California, 2022

**DOI:** 10.1093/infdis/jiad448

**Published:** 2023-10-13

**Authors:** Kristen A Wendorf, Rilene Ng, Cameron Stainken, Meredith Haddix, Erin Peterson, Jessica Watson, Darpun Sachdev

**Affiliations:** California Department of Public Health, Richmond, California, USA; California Department of Public Health, Richmond, California, USA; California Department of Public Health, Richmond, California, USA; Los Angeles County Department of Public Health, Los Angeles, California, USA; Los Angeles County Department of Public Health, Los Angeles, California, USA; California Department of Public Health, Richmond, California, USA; California Department of Public Health, Richmond, California, USA

**Keywords:** children, household transmission, mpox, pediatric, secondary attack rate

## Abstract

**Background:**

In California, the 2022 mpox outbreak cumulated 5572 cases, 20% of US cases, as of November 28, 2022; 0.3% of cases were among children <16 years old. The secondary attack rate (SAR) for children sharing households with infected adults is unknown.

**Methods:**

A line list of pediatric mpox household contacts aged <16 years reported through August 31, 2022 was created. It included demographic and clinical information on the contacts. Pediatric contact lists were crossmatched with the state vaccination database to identify those who received postexposure prophylaxis (PEP) with the JYNNEOS vaccine.

**Results:**

We identified 129 pediatric household contacts with median age of 7 years (range, 0–15 years). Among 18 symptomatic contacts, 12 (66.7%) underwent mpox testing; 5 (41.2%) were confirmed cases, 6 (50%) were negative, and 1 (0.8%) had an indeterminate result. Six symptomatic children were not tested for mpox (33.3%). Overall, 6 infected contacts were identified, resulting in a SAR of 4.7% (6 of 129). The majority of pediatric household contacts and 4 of 6 infected children identified as Hispanic/Latino. Only 18 children (14%) reported receiving PEP.

**Conclusions:**

The SAR was overall low among pediatric household contacts; none had severe disease. This may be underestimated given low testing rates.

The 2022 clade IIb mpox outbreak cumulated 5572 cases in California as of 28 November 2022, approximately 20% of the US case count [1]; 0.3% of cases were among children <16 years old [2]. Mpox is endemic in West and Central Africa with reported person-to-person transmission via direct contact with infection skin lesions, body fluids, or materials contaminated with infected body fluids [3]. In these prior outbreaks, mpox transmission in households has been described [4–6]. Previous studies have described risk factors of spread in households including sleeping in the same room or bed, sharing food from the same dish, and drinking out of the same cup as a primary case [6]. In a systematic review, the secondary attack rate (SAR) among unvaccinated household contacts ranged from 0% to 11%, with a pooled estimate of 8%, and the majority of transmission occurring from children to adults [7]. Finally, limited data from prior outbreaks also suggested that pediatric patients were more likely to be hospitalized in intensive care units compared to adults [8].

Early in the clade IIb outbreak in the United States, epidemiological risk factors for children and adolescents were unclear. Most (approximately 88%) of the early cases in California who reported their sexual orientation occurred among individuals who identified as gay, lesbian, bisexual, or other men who have sex with men, with frequent findings of lesions limited to the genital area and rectal pain. A variable temporal association was also observed between mucocutaneous and systemic features, both of which suggested new clinical and epidemiological spread for the disease [9]. Only a handful of cases had been reported in persons younger than 18 years old as of 31 August 2022; the majority of children <16 had a known adult source case, while most older teenagers reported potential exposures outside of the household. Given that prior outbreaks from sub-Saharan Africa suggested children in the household were at significant risk of illness and severe disease from mpox, and also able to transmit disease via close contact to other children, we sought to understand the risk of household transmission to children in the current outbreak, especially as cases were peaking at a time when children were returning to school after the summer break [8, 10].

To understand the risk of mpox transmission to pediatric contacts, we analyzed routinely collected contact tracing data in California to assess the SAR among pediatric household contacts to reported mpox cases. As part of this assessment, we also sought to understand the extent to which household pediatric contacts received postexposure prophylaxis (PEP) with the JYNNEOS vaccine.

## METHODS

Using the California public health database, we created a line list of all pediatric mpox contacts aged < 16 years reported through 31 August 2022. Contacts to a nonconfirmed or probable case of mpox and nonhousehold contacts were excluded from analysis. For this study, confirmed cases had identification of mpox virus DNA by molecular sequencing or isolation of mpox in culture of the patient's clinical specimen; probable cases had no suspicion for other recent orthopoxvirus exposure and had positive orthopoxvirus molecular findings, positive orthopoxvirus immunohistochemical findings, orthopoxvirus immunoglobulin M (IgM) antibodies presenting within 4–56 days of rash onset, or for young children without testing but known mpox household exposure and clinical signs and symptoms of mpox within 21 days of known exposure. Samples were considered indeterminate if no human Rnase P gene was detected.

Line lists with deidentified data were collected from Los Angeles and San Diego counties, who do not contribute to the state database. All pediatric contacts were reviewed to identify those who developed symptoms consistent with mpox during their incubation period (21 days after last exposure to mpox) and mpox testing that occurred; identified pediatric contacts that did not report symptoms were assumed to have been asymptomatic. Local public health departments asked caregivers to report any symptoms potentially consisted with mpox among pediatric household contacts during the incubation period. Children with reported skin lesions were offered mpox lesion-based testing, which were collected in accordance with Centers for Disease Control and Prevention (CDC) guidance and run on an mpox specific reverse transcription polymerase chain reaction (RT-PCR) assay at the California Department of Public Health or the Los Angeles County Public Health Laboratory. Some pediatric contacts who did not undergo lesion testing were later offered orthopoxvirus IgM antibody testing. Included pediatric household contacts were crossmatched with our state vaccination database to identify those who received PEP with the JYNNEOS vaccine.

## RESULTS

**Figure 1.**
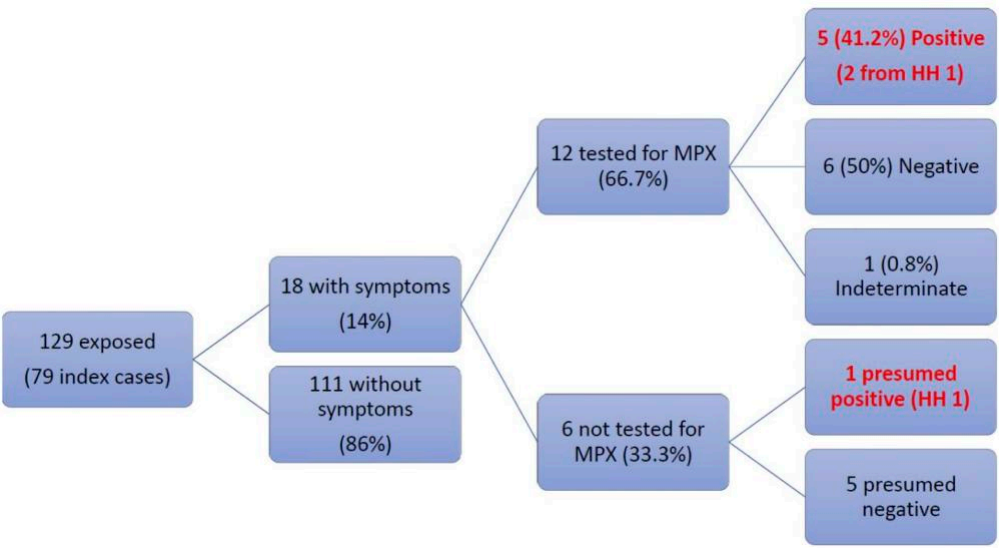
Outcomes of mpox surveillance among pediatric household contacts in California. Abbreviations: HH, household; MPX, mpox virus.

We identified 129 pediatric household contacts exposed by 79 index cases (Figure 1). The median age of contacts was 7 years (range, 0–15 years). Eighteen children (14%) exposed by 14 index cases reported symptoms consistent with mpox during their incubation period; their median age was 6 years (range, 1–11 years). All symptomatic children reported a rash; other symptoms included adenopathy, fever, fatigue, and myalgia.

Among 18 symptomatic contacts, 12 (66.7%) underwent mpox testing; 5 (41.2%) were positive and confirmed cases, 6 (50%) were negative, and 1 (0.8%) had an indeterminate result (repeat testing was also indeterminate and child was not thought to be a case based on clinical presentation and risk factors). Two of the confirmed infected pediatric contacts came from a single household (household 1). Among the symptomatic children who were tested and negative, all were believed to be true negatives. Two children had fevers and a handful of lesions on their faces and groins thought to be consistent with coxsackievirus infection. One child had a rash on the legs only with diarrhea; a photo of the rash was reviewed and not consistent with the pustules of mpox. Two other children denied systemic symptoms; 1 had a few nonpustule lesions on the arm thought to be bug bites, the other had lesions on the face thought to be acne. The final child who tested negative was reported to have a rash, but appearance of the rash or presence of other symptoms was unknown. Asymptomatic pediatric contacts were not tested for mpox.

Six symptomatic children who were household contacts were not tested for mpox (33.3%). One child, a resident of household 1, was presumed to have mpox based on a consistent rash and systemic symptoms; the child was not able to be tested during the acute phase and IgM testing was declined so this child was considered a probable case. Two children with skin lesions but no systemic symptoms were never tested; 1 had a single skin lesion and refused testing, the other had a rash felt not to be consistent with mpox after physician examination. It is unknown why testing did not occur for the remaining 3 children.

Six confirmed and probable cases of mpox were identified among pediatric household contacts in California through 31 August 2022. The median age of the secondary cases was 4.5 years (range, 2–9 years). Three of the 6 (50%) confirmed/probable cases resided in the same household (household 1). The SAR of mpox was 4.7% among pediatric household contacts all together, but 7.1% among those children aged 0–9 years (Table 1). All 6 of the pediatric cases had mild disease; none required hospitalization.

**Table 1.** Demographic and Clinical Characteristics of 129 Household Pediatric Contacts Exposed by 79 Adult Index Cases

Characteristic	Pediatric Household Contacts, No. (%)	Tested Contacts, No. (%)	Secondary Attack Rate, % (No./Total No.)
Race/ethnicity	129 (100)	12 (9)	4.7 (6/129)
Black/African American	5 (4)	1 (20)	20 (1/5)
Hispanic or Latino	80 (62)	7 (9)	5 (4/80)
White	23 (18)	1 (4)	0 (0/23)
Asian	4 (3)	2 (50)	0 (0/4)
Other/unknown	17 (13)	1 (6)	5.8 (1/17)
Age, years			
0–9	85 (66)	12 (14)	7.1 (6/85)
10–15	44 (33)	0 (0)	…
Received PEP	18 (14)	1 (6)	0 (0/18)

Abbreviation: PEP, postexposure prophylaxis or vaccination.

The majority of identified pediatric household contacts identified as Hispanic or Latino (62%) with 4 of the 6 cases (67%) identifying as Hispanic or Latino (Table 1). Only 18 children (14%) received PEP based on vaccine records. Among these vaccinated children only 1 was tested for mpox and that child tested negative.

## DISCUSSION

Among children experiencing close household contact with an mpox case in California, only 14% developed symptoms consistent with mpox, and less than 5% ultimately tested positive. Although most children exposed within the household were contacts to adult mpox cases, 1 of the confirmed cases in this series was exposed by an older sibling in the household. Three of 6 children who acquired mpox through household contact resided in the same household, suggesting factors specific to the index cases might have impacted transmission. In this household, the 2 index cases were male parenting adults, both with delayed diagnoses of mpox and prolonged period without precautions taken within the household, and with direct physical contact with all 3 children. In addition, another caregiver residing outside of this household also developed symptoms and tested positive for mpox.

Overall, infected pediatric contacts tended to be younger than uninfected contacts, suggesting more intimate contact with activities of daily living might have increased risk for transmission. The SAR was 7.1% for children <9 years old and dropped to 0% for pediatric household contacts aged 10 or older. Only 14% of pediatric contacts received PEP, despite the CDC recommendation for all contacts aged 6 months or older be vaccinated soon after exposure.

Our study had several limitations. First, the disease investigation follow-up of exposed pediatric contacts varied among counties within CA, so the symptom screening frequency was unknown, and we had to presume that the lack of report of symptoms meant the child had no symptoms. Testing was limited to children with skin lesions; 1 child underwent confirmatory IgM testing, but others who were offered this testing declined. Given the use of surveillance data and the limitations of mpox lesion-based testing, we would not have been able to detect children with asymptomatic infection or without skin lesions, as well as those who declined testing.

While this is one of the initial reports during the 2022 outbreak to assess household transmission of mpox to children, our results are consistent with observational data reported as part of published case series [10, 11]. Our data further support that close, direct physical contact is the predominant mode of transmission in the 2022 clade IIb outbreak, given the low observed SAR to children within households. Monitoring of pediatric contacts for symptoms is warranted following potential household exposure of mpox, but overall, the risk of transmission to pediatric contacts appears low.

## Notes

***Author contributions.*** K. A. W. designed the study, performed the primary analysis, drafted the initial manuscript, and critically reviewed and revised the manuscript. R. N. collected the data and created line lists and critically reviewed and revised the manuscript. C. S. and J. W. critically reviewed and revised the manuscript. M. H. and E. P. collated data and reviewed the manuscript. D. S. conceptualized and designed the project, and critically reviewed and revised the manuscript. All authors approved the final manuscript as submitted and agree to be accountable for all aspects of the work.

***Acknowledgments.*** Thank you to all the counties in California who contributed data to this analysis. Special thanks to San Diego and Los Angeles Counties for providing line lists to include with our data as well as Fresno, Contra Costa, Merced, Stanislaus, and Sacramento counties who provided follow-up data.

***Financial support.*** No financial support was received for this work.

***Supplement sponsorship***. This article appears as part of the supplement “Mpox: Challenges and Opportunities Following the Global 2022 Outbreak,” sponsored by the Centers for Disease Control and Prevention (Atlanta, GA).
